# 1-Benzyl-2-phenyl-1*H*-benzimidazole

**DOI:** 10.1107/S1600536809001238

**Published:** 2009-01-14

**Authors:** Lingqian Kong

**Affiliations:** a Dongchang College, Liaocheng University, Liaocheng 250059, People’s Republic of China

## Abstract

The title compound, C_20_H_16_N_2_, has been synthesized by the reaction of benzaldehyde with *o*-phenyl­endiamine and l-proline. The benzimidazole group makes a dihedral angle of 29.04 (1)° with the attached benzene ring, and is approximately perpendicular to the plane of the benzyl group [dihedral angle = 88.9 (1)°] The crystal packing exhibits no unusually short inter­molecular contacts.

## Related literature

For background literature concerning benzimidazole compounds, see: Zarrinmayeh *et al.* (1998 ▶); Spasov *et al.* (1999 ▶). For a related structure, see: Yang *et al.* (2007 ▶).
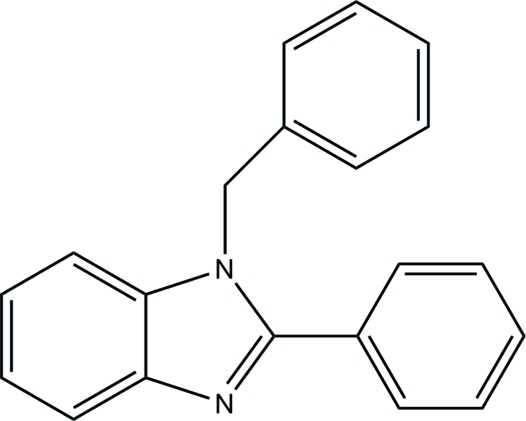


## Experimental

### 

#### Crystal data

C_20_H_16_N_2_
*M* *_r_* = 284.35
Orthorhombic, 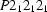

*a* = 6.338 (3) Å
*b* = 8.085 (3) Å
*c* = 30.190 (12) Å
*V* = 1547.0 (10) Å^3^
*Z* = 4
Mo *K*α radiation
μ = 0.07 mm^−1^
*T* = 298 (2) K
0.63 × 0.55 × 0.47 mm

#### Data collection

Bruker SMART CCD diffractometer
Absorption correction: multi-scan (*SADABS*; Sheldrick, 1996 ▶) *T*_min_ = 0.956, *T*_max_ = 0.967
6729 measured reflections
1631 independent reflections
1221 reflections with *I* > 2σ(*I*)
*R*_int_ = 0.073

#### Refinement

*R*[*F* ^2^ > 2σ(*F* ^2^)] = 0.045
*wR*(*F* ^2^) = 0.115
*S* = 1.14
1631 reflections
199 parameters
H-atom parameters constrained
Δρ_max_ = 0.14 e Å^−3^
Δρ_min_ = −0.16 e Å^−3^

### 

Data collection: *SMART* (Bruker, 1997 ▶); cell refinement: *SAINT* (Bruker, 1997 ▶); data reduction: *SAINT*; program(s) used to solve structure: *SHELXS97* (Sheldrick, 2008 ▶); program(s) used to refine structure: *SHELXL97* (Sheldrick, 2008 ▶); molecular graphics: *SHELXTL* (Sheldrick, 2008 ▶); software used to prepare material for publication: *SHELXTL*.

## Supplementary Material

Crystal structure: contains datablocks I, global. DOI: 10.1107/S1600536809001238/bi2334sup1.cif

Structure factors: contains datablocks I. DOI: 10.1107/S1600536809001238/bi2334Isup2.hkl

Additional supplementary materials:  crystallographic information; 3D view; checkCIF report

## Figures and Tables

**Fig. 1.**
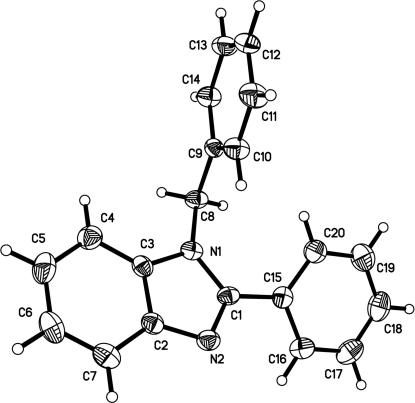
Molecular structure showing 30% probability displacement ellipsoids for non-H atoms.

## supplementary crystallographic information

### Comment

The benzimidazole group is of significant importance in medicinal chemistry. Several publications report benzimidazole-containing compounds showing biological activities such as selective neuropeptide receptor antagonism (Zarrinmayeh, *et al.*,1998). Substituted benzimidazole derivatives have found commercial applications in veterinary medicine as anthelmintic agents and in diverse human therapeutic areas such as treatment of ulcers and as antihistaminics (Spasov, *et al.*,1999).

In the crystal structure of the title compound, the imidazole ring is almost coplanar with the benzene ring (C2/C3/C4/C5/C6/C7): the C1—N1—C3—C2 and C1—N2—C2—C3 torsion angles are 0.0 (3)° and -0.8 (3)°, respectively. The dihedral angles between the imidazole ring and the benzene rings (C2/C3/C4/C5/C6/C7) and (C15/C16/C17/C18/C19/C20) are 2.84 (1)° and 29.54 (1)°, respectively. There are no significantly short intermolecular contacts.

### Experimental

*o*-Phenylendiamine (5 mmol), benzaldehyde (10 mmol), *L*-proline (1 mmol) and 10 ml ethanol were mixed in a 50 ml flask. After stirring for 4 h at 373 K, the resulting mixture was recrystalized from ethanol, affording the title compound as an orange crystalline solid. Elemental analysis calculated: C 84.48, H 5.67, N 9.85%; found: C 84.38, H 5.54, N 9.77%.

### Refinement

H atoms were placed in geometrically idealized positions (methylene C—H = 0.97 Å, aromatic C—H = 0.93 Å) and treated as riding on their parent atoms, with *U*_iso_(H) = 1.2*U*_eq_(C). In the absence of significant anomalous scattering, Friedel pairs have been merged as equivalent data.

### Crystal data

**Table:**

C_20_H_16_N_2_	*F*(000) = 600
*M**_r_* = 284.35	*D*_x_ = 1.221 Mg m^−^^3^
Orthorhombic, *P*2_1_2_1_2_1_	Mo *K*α radiation, λ = 0.71073 Å
Hall symbol: P 2ac 2ab	Cell parameters from 1704 reflections
*a* = 6.338 (3) Å	θ = 2.6–21.8°
*b* = 8.085 (3) Å	µ = 0.07 mm^−^^1^
*c* = 30.190 (12) Å	*T* = 298 K
*V* = 1547.0 (10) Å^3^	Block, orange
*Z* = 4	0.63 × 0.55 × 0.47 mm

### Data collection

**Table:**

Bruker SMART CCD diffractometer	1631 independent reflections
Radiation source: fine-focus sealed tube	1221 reflections with *I* > 2σ(*I*)
graphite	*R*_int_ = 0.073
φ and ω scans	θ_max_ = 25.1°, θ_min_ = 1.4°
Absorption correction: multi-scan (*SADABS*; Sheldrick, 1996)	*h* = −7→7
*T*_min_ = 0.956, *T*_max_ = 0.967	*k* = −9→7
6729 measured reflections	*l* = −26→36

### Refinement

**Table:**

Refinement on *F*^2^	Primary atom site location: structure-invariant direct methods
Least-squares matrix: full	Secondary atom site location: difference Fourier map
*R*[*F*^2^ > 2σ(*F*^2^)] = 0.045	Hydrogen site location: inferred from neighbouring sites
*wR*(*F*^2^) = 0.115	H-atom parameters constrained
*S* = 1.14	*w* = 1/[σ^2^(*F*_o_^2^) + (0.0509*P*)^2^] where *P* = (*F*_o_^2^ + 2*F*_c_^2^)/3
1631 reflections	(Δ/σ)_max_ < 0.001
199 parameters	Δρ_max_ = 0.14 e Å^−^^3^
0 restraints	Δρ_min_ = −0.16 e Å^−^^3^

### Special details

**Table:**

Geometry. All e.s.d.'s (except the e.s.d. in the dihedral angle between two l.s. planes) are estimated using the full covariance matrix. The cell e.s.d.'s are taken into account individually in the estimation of e.s.d.'s in distances, angles and torsion angles; correlations between e.s.d.'s in cell parameters are only used when they are defined by crystal symmetry. An approximate (isotropic) treatment of cell e.s.d.'s is used for estimating e.s.d.'s involving l.s. planes.
Refinement. Refinement of *F*^2^ against ALL reflections. The weighted *R*-factor *wR* and goodness of fit *S* are based on *F*^2^, conventional *R*-factors *R* are based on *F*, with *F* set to zero for negative *F*^2^. The threshold expression of *F*^2^ > σ(*F*^2^) is used only for calculating *R*-factors(gt) *etc*. and is not relevant to the choice of reflections for refinement. *R*-factors based on *F*^2^ are statistically about twice as large as those based on *F*, and *R*- factors based on ALL data will be even larger.

### Fractional atomic coordinates and isotropic or equivalent isotropic displacement parameters (Å^2^)

**Table:**

	*x*	*y*	*z*	*U*_iso_*/*U*_eq_	
N1	0.2537 (4)	−0.0386 (3)	0.13584 (8)	0.0404 (6)	
N2	0.5764 (4)	−0.1478 (3)	0.14294 (9)	0.0457 (7)	
C1	0.4421 (5)	−0.0765 (3)	0.11598 (10)	0.0383 (7)	
C2	0.4713 (5)	−0.1607 (3)	0.18323 (11)	0.0421 (8)	
C3	0.2703 (5)	−0.0923 (3)	0.17932 (10)	0.0406 (7)	
C4	0.1274 (6)	−0.0905 (4)	0.21384 (12)	0.0543 (9)	
H4	−0.0051	−0.0422	0.2110	0.065*	
C5	0.1920 (7)	−0.1639 (5)	0.25254 (13)	0.0678 (11)	
H5	0.1002	−0.1667	0.2766	0.081*	
C6	0.3909 (7)	−0.2340 (5)	0.25674 (13)	0.0683 (12)	
H6	0.4288	−0.2824	0.2835	0.082*	
C7	0.5324 (7)	−0.2341 (4)	0.22272 (12)	0.0588 (10)	
H7	0.6651	−0.2816	0.2259	0.071*	
C8	0.0616 (5)	0.0304 (4)	0.11706 (11)	0.0422 (8)	
H8A	−0.0587	−0.0290	0.1290	0.051*	
H8B	0.0630	0.0134	0.0853	0.051*	
C9	0.0341 (5)	0.2130 (3)	0.12639 (10)	0.0352 (7)	
C10	0.1930 (5)	0.3114 (4)	0.14264 (11)	0.0484 (9)	
H10	0.3250	0.2656	0.1482	0.058*	
C11	0.1592 (6)	0.4782 (4)	0.15079 (13)	0.0574 (10)	
H11	0.2680	0.5430	0.1620	0.069*	
C12	−0.0329 (6)	0.5478 (4)	0.14239 (12)	0.0551 (9)	
H12	−0.0550	0.6597	0.1477	0.066*	
C13	−0.1927 (6)	0.4519 (4)	0.12605 (12)	0.0559 (10)	
H13	−0.3239	0.4988	0.1203	0.067*	
C14	−0.1601 (5)	0.2856 (4)	0.11803 (11)	0.0479 (9)	
H14	−0.2698	0.2215	0.1069	0.057*	
C15	0.4924 (5)	−0.0445 (4)	0.06922 (10)	0.0422 (8)	
C16	0.6356 (5)	−0.1483 (4)	0.04824 (12)	0.0538 (9)	
H16	0.6957	−0.2351	0.0640	0.065*	
C17	0.6904 (6)	−0.1255 (6)	0.00461 (13)	0.0721 (12)	
H17	0.7854	−0.1972	−0.0089	0.087*	
C18	0.6052 (7)	0.0028 (5)	−0.01897 (14)	0.0737 (13)	
H18	0.6398	0.0174	−0.0487	0.088*	
C19	0.4695 (7)	0.1087 (5)	0.00140 (12)	0.0730 (12)	
H19	0.4147	0.1976	−0.0144	0.088*	
C20	0.4119 (6)	0.0867 (4)	0.04484 (11)	0.0555 (9)	
H20	0.3183	0.1603	0.0580	0.067*	

### Atomic displacement parameters (Å^2^)

**Table:**

	*U*^11^	*U*^22^	*U*^33^	*U*^12^	*U*^13^	*U*^23^
N1	0.0385 (15)	0.0347 (14)	0.0479 (17)	0.0033 (12)	−0.0049 (14)	−0.0007 (12)
N2	0.0378 (14)	0.0455 (16)	0.0537 (17)	0.0033 (13)	−0.0038 (14)	0.0020 (13)
C1	0.0364 (17)	0.0306 (16)	0.0480 (18)	0.0017 (14)	−0.0020 (16)	−0.0081 (13)
C2	0.0455 (19)	0.0329 (16)	0.048 (2)	−0.0025 (15)	−0.0023 (17)	−0.0001 (14)
C3	0.0487 (19)	0.0315 (16)	0.0416 (19)	−0.0025 (15)	−0.0015 (16)	0.0004 (14)
C4	0.058 (2)	0.051 (2)	0.054 (2)	0.0026 (18)	0.0044 (19)	−0.0065 (18)
C5	0.082 (3)	0.075 (3)	0.047 (2)	−0.008 (3)	0.014 (2)	−0.0034 (19)
C6	0.087 (3)	0.070 (3)	0.048 (2)	−0.006 (2)	−0.012 (2)	0.0142 (19)
C7	0.062 (2)	0.054 (2)	0.061 (2)	0.0028 (19)	−0.012 (2)	0.0057 (18)
C8	0.0329 (16)	0.0359 (16)	0.058 (2)	0.0006 (13)	−0.0057 (15)	−0.0050 (14)
C9	0.0362 (16)	0.0308 (15)	0.0386 (16)	−0.0016 (13)	0.0000 (15)	0.0013 (12)
C10	0.0438 (19)	0.0394 (19)	0.062 (2)	−0.0019 (15)	−0.0084 (18)	−0.0063 (15)
C11	0.062 (2)	0.037 (2)	0.073 (3)	−0.0080 (18)	−0.009 (2)	−0.0133 (17)
C12	0.064 (2)	0.0332 (18)	0.068 (2)	0.0079 (18)	0.006 (2)	−0.0025 (16)
C13	0.048 (2)	0.048 (2)	0.072 (2)	0.0140 (17)	0.000 (2)	0.0042 (18)
C14	0.0406 (19)	0.0438 (19)	0.059 (2)	0.0001 (15)	−0.0070 (17)	−0.0045 (16)
C15	0.0456 (19)	0.0395 (16)	0.0415 (18)	−0.0051 (16)	0.0004 (15)	−0.0052 (14)
C16	0.051 (2)	0.058 (2)	0.052 (2)	0.0088 (18)	−0.0033 (19)	−0.0058 (18)
C17	0.066 (3)	0.085 (3)	0.066 (3)	0.009 (2)	0.015 (2)	−0.018 (2)
C18	0.082 (3)	0.095 (3)	0.045 (2)	−0.008 (3)	0.015 (2)	−0.002 (2)
C19	0.084 (3)	0.081 (3)	0.054 (2)	0.003 (3)	−0.001 (2)	0.014 (2)
C20	0.060 (2)	0.056 (2)	0.051 (2)	0.0035 (18)	0.0035 (19)	0.0005 (17)

### Geometric parameters (Å, °)

**Table:**

N1—C1	1.371 (4)	C10—C11	1.387 (5)
N1—C3	1.387 (4)	C10—H10	0.930
N1—C8	1.454 (4)	C11—C12	1.365 (5)
N2—C1	1.311 (4)	C11—H11	0.930
N2—C2	1.391 (4)	C12—C13	1.368 (5)
C1—C15	1.470 (4)	C12—H12	0.930
C2—C7	1.387 (4)	C13—C14	1.382 (4)
C2—C3	1.394 (4)	C13—H13	0.930
C3—C4	1.381 (4)	C14—H14	0.930
C4—C5	1.373 (5)	C15—C20	1.388 (4)
C4—H4	0.930	C15—C16	1.389 (4)
C5—C6	1.388 (5)	C16—C17	1.375 (5)
C5—H5	0.930	C16—H16	0.930
C6—C7	1.363 (5)	C17—C18	1.369 (6)
C6—H6	0.930	C17—H17	0.930
C7—H7	0.930	C18—C19	1.360 (6)
C8—C9	1.513 (4)	C18—H18	0.930
C8—H8A	0.970	C19—C20	1.373 (5)
C8—H8B	0.970	C19—H19	0.930
C9—C10	1.374 (4)	C20—H20	0.930
C9—C14	1.387 (4)		
			
C1—N1—C3	106.1 (2)	C9—C10—C11	120.8 (3)
C1—N1—C8	130.1 (3)	C9—C10—H10	119.6
C3—N1—C8	123.6 (3)	C11—C10—H10	119.6
C1—N2—C2	105.4 (3)	C12—C11—C10	120.4 (3)
N2—C1—N1	113.1 (3)	C12—C11—H11	119.8
N2—C1—C15	122.2 (3)	C10—C11—H11	119.8
N1—C1—C15	124.7 (3)	C11—C12—C13	119.6 (3)
C7—C2—C3	119.8 (3)	C11—C12—H12	120.2
C7—C2—N2	130.6 (3)	C13—C12—H12	120.2
C3—C2—N2	109.5 (3)	C12—C13—C14	120.3 (3)
C4—C3—N1	131.4 (3)	C12—C13—H13	119.9
C4—C3—C2	122.7 (3)	C14—C13—H13	119.9
N1—C3—C2	105.9 (3)	C13—C14—C9	120.8 (3)
C5—C4—C3	116.2 (4)	C13—C14—H14	119.6
C5—C4—H4	121.9	C9—C14—H14	119.6
C3—C4—H4	121.9	C20—C15—C16	117.4 (3)
C4—C5—C6	121.7 (4)	C20—C15—C1	124.3 (3)
C4—C5—H5	119.2	C16—C15—C1	118.2 (3)
C6—C5—H5	119.2	C17—C16—C15	121.4 (3)
C7—C6—C5	121.9 (4)	C17—C16—H16	119.3
C7—C6—H6	119.0	C15—C16—H16	119.3
C5—C6—H6	119.0	C18—C17—C16	120.0 (4)
C6—C7—C2	117.6 (4)	C18—C17—H17	120.0
C6—C7—H7	121.2	C16—C17—H17	120.0
C2—C7—H7	121.2	C19—C18—C17	119.4 (4)
N1—C8—C9	113.4 (2)	C19—C18—H18	120.3
N1—C8—H8A	108.9	C17—C18—H18	120.3
C9—C8—H8A	108.9	C18—C19—C20	121.2 (4)
N1—C8—H8B	108.9	C18—C19—H19	119.4
C9—C8—H8B	108.9	C20—C19—H19	119.4
H8A—C8—H8B	107.7	C19—C20—C15	120.5 (3)
C10—C9—C14	118.1 (3)	C19—C20—H20	119.7
C10—C9—C8	123.2 (3)	C15—C20—H20	119.7
C14—C9—C8	118.8 (3)		
			
C2—N2—C1—N1	0.9 (3)	C3—N1—C8—C9	83.9 (3)
C2—N2—C1—C15	−178.4 (3)	N1—C8—C9—C10	12.9 (4)
C3—N1—C1—N2	−0.6 (3)	N1—C8—C9—C14	−167.4 (3)
C8—N1—C1—N2	−175.6 (3)	C14—C9—C10—C11	0.6 (5)
C3—N1—C1—C15	178.6 (3)	C8—C9—C10—C11	−179.7 (3)
C8—N1—C1—C15	3.6 (5)	C9—C10—C11—C12	−0.6 (6)
C1—N2—C2—C7	175.9 (3)	C10—C11—C12—C13	0.3 (6)
C1—N2—C2—C3	−0.8 (3)	C11—C12—C13—C14	−0.1 (6)
C1—N1—C3—C4	−177.9 (3)	C12—C13—C14—C9	0.1 (5)
C8—N1—C3—C4	−2.4 (5)	C10—C9—C14—C13	−0.3 (5)
C1—N1—C3—C2	0.0 (3)	C8—C9—C14—C13	180.0 (3)
C8—N1—C3—C2	175.5 (2)	N2—C1—C15—C20	−149.6 (3)
C7—C2—C3—C4	1.6 (4)	N1—C1—C15—C20	31.2 (5)
N2—C2—C3—C4	178.6 (3)	N2—C1—C15—C16	28.0 (4)
C7—C2—C3—N1	−176.6 (3)	N1—C1—C15—C16	−151.2 (3)
N2—C2—C3—N1	0.5 (3)	C20—C15—C16—C17	−2.1 (5)
N1—C3—C4—C5	176.1 (3)	C1—C15—C16—C17	−179.9 (3)
C2—C3—C4—C5	−1.5 (5)	C15—C16—C17—C18	0.7 (6)
C3—C4—C5—C6	0.8 (5)	C16—C17—C18—C19	1.2 (7)
C4—C5—C6—C7	−0.1 (6)	C17—C18—C19—C20	−1.8 (6)
C5—C6—C7—C2	0.1 (5)	C18—C19—C20—C15	0.4 (6)
C3—C2—C7—C6	−0.8 (5)	C16—C15—C20—C19	1.5 (5)
N2—C2—C7—C6	−177.2 (3)	C1—C15—C20—C19	179.2 (3)
C1—N1—C8—C9	−101.8 (4)		
